# Synthesis and crystal structure of bis(1-{[(quinolin-8-yl)imino]methyl}pyrene-κ^2^ *N*,*N*′)silver(I) tri­fluoro­methane­sulfonate

**DOI:** 10.1107/S205698901601519X

**Published:** 2016-09-30

**Authors:** Miguel Pinto, Indranil Chakraborty, Pradip Mascharak

**Affiliations:** a Department of Chemistry, University of California Santa Cruz, CA 95064

**Keywords:** crystal structure, pyrene, π-stacking, anti­microbial, silver complex

## Abstract

The title compound, [Ag(qPyr)_2_]CF_3_SO_3_ where qPyr = 1-(quinoline-2-yl­methyl­ene)amino­pyrene, C_26_H_16_N_2_, was synthesized from a reaction of silver tri­fluoro­methane­sulfonate and qPyr in di­chloro­methane–methanol mixed media. In this design, the qPyr ligand was chosen for its characteristic excitation and emission profiles, which could enable the tracking of the silver complex within biological targets. The Ag^I^ atom resides in a distorted tetra­hedral N_4_ coordination sphere. Analysis of the packing pattern revealed significant intra- and inter­molecular π–π stacking inter­actions between the [Ag(qPyr)_2_]^+^ cations. In addition, a weak C—H⋯O hydrogen bond consolidates the packing between cations and anions.

## Abstract 2

In the title salt, [Ag(qPyr)_2_]CF_3_SO_3_ where qPyr = 1-(quinoline-2-yl­methyl­ene)amino­pyrene, the Ag^I^ atom exhibits a distorted tetra­hedral coordination by two chelating 1-(quinoline-2-yl­methyl­ene)amino­pyrene ligands.

## Chemical context   

Silver metal and its salts have been used for their well known anti­microbial properties since ancient times (Chernousova & Epple, 2013 ▸). In recent years, the use of silver has regained inter­est due to the emergence of multidrug-resistant organisms (MDROs) (Kresse *et al.*, 2007 ▸; Liu *et al.*, 2010 ▸; Thornton *et al.*, 2016 ▸). Silver is primarily used topically to treat chronic infections in burn wounds (deBoer *et al.*, 2015 ▸). The metal exerts its microbial toxicity by slowly releasing Ag^I^ ions that inflict damage on cell walls, produce reactive oxygen species and bind to DNA base pairs as well as proteins, impeding normal cellular functions (Liu *et al.*, 2010 ▸; Thornton *et al.*, 2016 ▸). As silver ions tend to precipitate as AgCl in the presence of blood plasma chloride (Chernousova & Epple, 2013 ▸), there is a need for stable silver complexes that can slowly and sustainably release silver ions into biological matrices. Herein we report the synthesis and characterization of a novel silver complex, [Ag(qPyr)_2_]CF_3_SO_3_ [where qPyr = 1-(quinoline-2-yl­methyl­ene)amino­pyrene] which could serve as a stable complex for the delivery of silver. In the design of this compound, qPyr was included due to its characteristic absorption and emission profile, which could allow tracking of the ligand and silver within the cell membrane of the bacteria (Ray *et al.*, 2006 ▸).

## Structural commentary   

**Figure 1:**
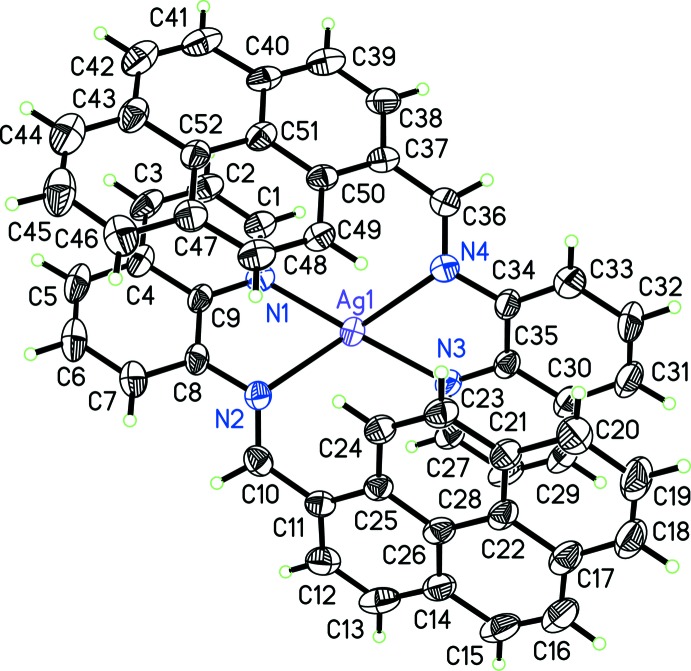
The mol­ecular structure of the cation in the title salt. Displacement ellipsoids correspond to the 50% probability level; the counter-anion is not shown.

The mol­ecular structure of the cation in the title complex is shown in Fig. 1 ▸. The coordination environment of the Ag^I^ atom in the cationic complex is distorted tetra­hedral (Table 1 ▸). The qPyr ligand binds to the metal in a bidentate fashion. In this complex, the chelate rings composed of atoms Ag1, N2, C8, C9, N1 and Ag1, N4, C34, C35, N3 are reasonably planar, with mean deviations of 0.054 (3) and 0.059 (3) Å, respectively. The dihedral angle between these two chelate planes is 69.0 (4)°. The two quinoline fragments within the qPyr ligand in the title complex are satisfactorily planar, with mean deviations of 0.031 (4) and 0.035 (4) Å. The dihedral angles between the quinoline moieties and the pyrene rings are quite similar [73.5 (4) and 73.8 (3)°].
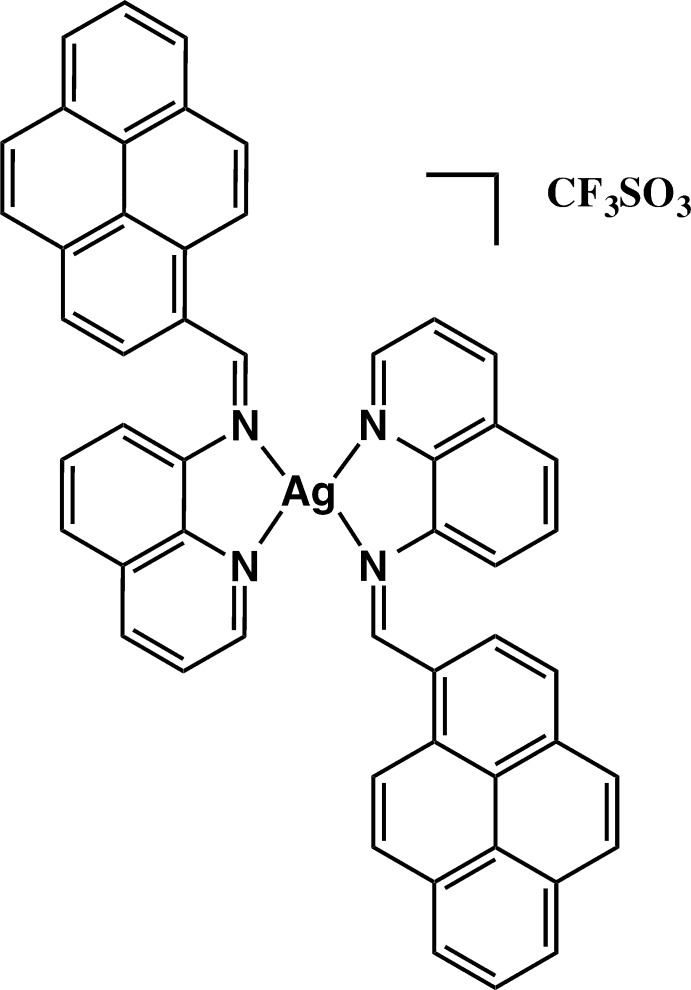


## Supra­molecular features   

**Figure 2:**
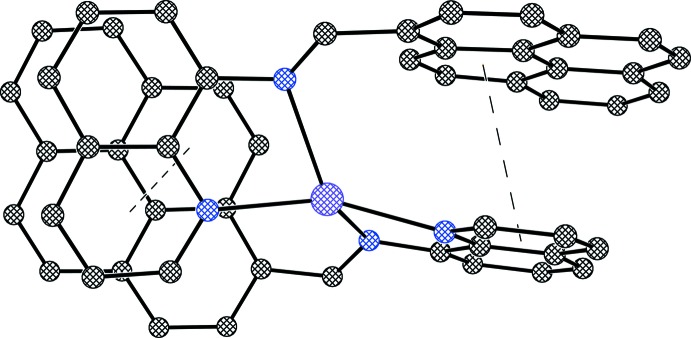
Representation of intra­molecular π–π stacking within the title complex.

**Figure 4:**
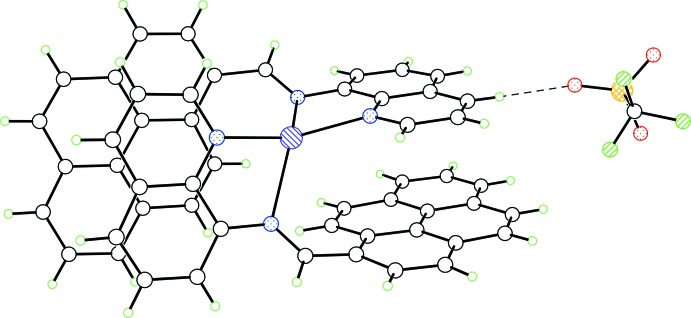
Packing pattern of the title salt showing the C—H⋯O inter­action between cation and anion.

**Figure 5:**
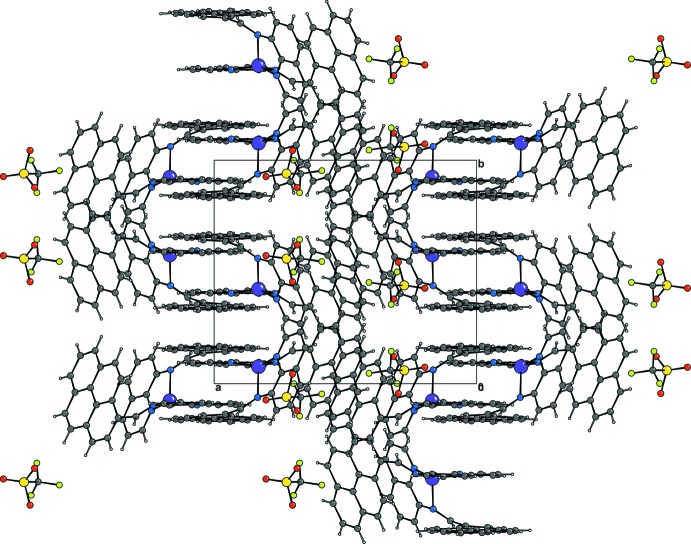
Packing diagram of the title salt along the *c* axis.

The packing pattern exhibits the presence of both intra- and inter­molecular offset π–π stacking inter­actions (Figs. 2 ▸ and 3 ▸). The extent of the inter­molecular π–π inter­action is found to be relatively stronger [3.543 (5) Å] compared to the intra­molecular π–π stacking inter­actions [3.642 (5) and 3.617 (5) Å]. In both cases, the angle between the ring normal and the vector between the ring centroids is close to 20° and centroid-to-centroid distances are within the upper limit of 3.8 Å (Janiak, 2000 ▸). The crystal packing of the complex reveals also a non-classical hydrogen-bonding inter­action (Steiner, 1996 ▸) of the type C—H⋯O between the cation and the triflate anion (Table 2 ▸, Fig. 4 ▸). The arrangement of the two types of mol­ecules along the *c* axis is shown in Fig. 5 ▸.

## Database survey   

A search of Cambridge Structural Database (Groom *et al.*, 2016 ▸) revealed that mol­ecular systems where Ag^I^ resides in a distorted tetra­hedral coordination environment are primarily of a supra­molecular nature. In a relatively recent report, two discrete Ag complexes, namely [Ag(H*L* ^1^)_2_](PF_6_) and [Ag(H*L* ^1^)_2_](NO_3_)(H_2_O) (where H*L* ^1^ = (*n*-Py)—CH=N—C_10_H_6_–COOH) are reported (Lee & Lee, 2013 ▸) which are structurally similar to the title complex. Both these mol­ecules adopt triclinic symmetry in space group *P* 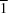. The average Ag—N distances for these complexes are slightly longer (2.349 and 2.346 Å) than that of the title complex (2.322 Å). Unlike the present complex, these two Ag complexes are characterized by significant intra­molecular O—H⋯F and O—H⋯O hydrogen-bonding inter­actions with the PF_6_^−^ and NO_3_^−^ counter-ions. In another report, three Ag complexes, namely [Ag(**1**)_2_](NO_3_), [Ag(**1**)_2_](PF_6_) and [Ag(**1**)_2_](OTf) [where **1** = (*R*)-2-(pyridin-2-yl­methyl­imino)-2′-(di­methyl­amino)-1,1′-binapth­yl] are described with similar structural features (Zhang *et al.*, 2011 ▸). In this study, [Ag(**1**)_2_](NO_3_) and [Ag(**1**)_2_](PF_6_) crystallize in space group *P*2_1_2_1_2_1_ while [Ag(**1**)_2_](OTf) crystallizes in *P*2_1_. Here the Ag—N distances span the range 2.354–2.376 Å, noticeably longer than that of the title complex.

## Synthesis and crystallization   

**Synthesis of the qPyr ligand**

A solution of 1-pyrenecarboxaldehyde (115 mg, 0.50 mmol) in 10 ml of di­chloro­methane was added drop wise to a solution of 8-amino­quinoline (72 mg, 0.50 mmol) in 10 ml of methanol. The mixture was heated to reflux for 16 h and then concentrated under reduced pressure. The precipitate thus formed was collected by vacuum filtration affording 162 mg (91% yield) of *N*-(1-pyrene)-1-quinolin-2-ylmethanimine (qPyr) as a light-brown powder.

**Synthesis of the title complex**

Two equivalents of qPyr (100 mg, 0.28 mmol) were dissolved in 20 ml of 1:1 methanol:di­chloro­methane along with one equivalent of silver tri­fluoro­methane­sulfonate (36 mg, 0.14 mmol). The reaction mixture was then stirred for 12 h. After this time, the solution was concentrated under reduced pressure. The resulting precipitate was collected through vacuum filtration affording a light-yellow powder. This powder was recrystallized from methanol to obtain [Ag(qPyr)_2_]CF_3_SO_3_ as a light yellow–brown powder (124 mg, 91%). Single crystals were obtained by vapor diffusion of ethyl ether into a solution of [Ag(qPyr)_2_]CF_3_SO_3_ in methanol.

## Refinement   

Crystal data, data collection and structure refinement details are summarized in Table 3 ▸. Hydrogen atoms were included in calculated positions on the C atoms to which they are bonded, with C—H = 0.93 Å and *U*_iso_(H) = 1.2*U*_eq_(C).

## Supplementary Material

Crystal structure: contains datablock(s) I. DOI: 10.1107/S205698901601519X/wm5314sup1.cif

Structure factors: contains datablock(s) I. DOI: 10.1107/S205698901601519X/wm5314Isup2.hkl

CCDC reference: 1506793

Additional supporting information: crystallographic information; 3D view; checkCIF report

## Figures and Tables

**Figure 3:**
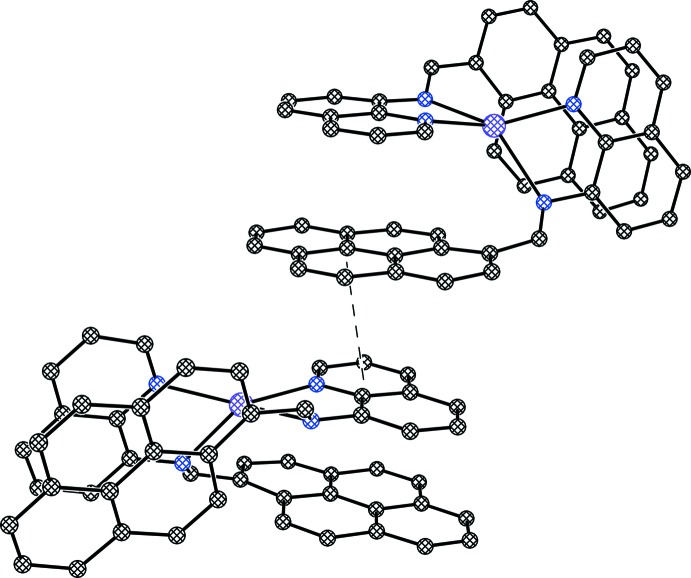
Representation of inter­molecular π–π stacking within the title complex.

**Table 1:** Selected geometric parameters (Å, °)

Ag1—N3	2.249 (4)	Ag1—N4	2.411 (4)
Ag1—N1	2.228 (4)	Ag1—N2	2.399 (4)
			
N3—Ag1—N4	72.24 (15)	N1—Ag1—N4	119.33 (15)
N3—Ag1—N2	120.45 (15)	N1—Ag1—N2	73.09 (15)
N1—Ag1—N3	151.52 (16)	N2—Ag1—N4	131.88 (14)

**Table 2:** Hydrogen-bond geometry (Å, °)

*D*—H⋯*A*	*D*—H	H⋯*A*	*D*⋯*A*	*D*—H⋯*A*
C3—H3⋯O3^i^	0.93	2.44	3.359 (10)	140

Symmetry code: (i) 
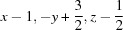
.

**Table 3:** Experimental details

Crystal data	
Chemical formula	[Ag(C_26_H_16_N_2_)_2_]CF_3_SO_3_
*M*_r_	969.75
Crystal system, space group	Monoclinic, *P*2_1_/*c*
Temperature (K)	273
*a*, *b*, *c* (Å)	17.132 (1), 13.6108 (8), 18.9712 (11)
β (°)	110.887 (1)
*V* (Å^3^)	4133.0 (4)
*Z*	4
Radiation type	Mo *K*α
μ (mm^−1^)	0.61
Crystal size (mm)	0.15 × 0.07 × 0.03

Data collection	
Diffractometer	Bruker APEXII CCD
Absorption correction	Multi-scan (*SADABS*; Bruker, 2012 ▸)
*T*_min_, *T*_max_	0.627, 0.745
No. of measured, independent and observed [*I* > 2σ(*I*)] reflections	35302, 7406, 4227
*R*_int_	0.084
(sin θ/λ)_max_ (Å^−1^)	0.602

Refinement	
*R*[*F* ^2^ > 2σ(*F* ^2^)], *wR*(*F* ^2^), *S*	0.058, 0.181, 1.03
No. of reflections	7406
No. of parameters	587
No. of restraints	598
H-atom treatment	H-atom parameters constrained
Δρ_max_, Δρ_min_ (e Å^−3^)	0.60, −0.54

Computer programs: *APEX2* and *SAINT* (Bruker, 2012 ▸), *SHELXT2014* (Sheldrick, 2015*a* ▸), *SHELXL2014* (Sheldrick, 2015*b* ▸), *XP* in *SHELXTL* (Sheldrick, 2008 ▸), *CrystalMaker* (Palmer, 2014 ▸) and *OLEX2* (Dolomanov *et al.*, 2009 ▸).

## supplementary crystallographic information

### Crystal data

**Table:**

[Ag(C_26_H_16_N_2_)_2_]CF_3_SO_3_	*F*(000) = 1968
*M**_r_* = 969.75	*D*_x_ = 1.558 Mg m^−^^3^
Monoclinic, *P*2_1_/*c*	Mo *K*α radiation, λ = 0.71073 Å
*a* = 17.132 (1) Å	Cell parameters from 3637 reflections
*b* = 13.6108 (8) Å	θ = 2.5–18.3°
*c* = 18.9712 (11) Å	µ = 0.61 mm^−^^1^
β = 110.887 (1)°	*T* = 273 K
*V* = 4133.0 (4) Å^3^	Block, yellow
*Z* = 4	0.15 × 0.07 × 0.03 mm

### Data collection

**Table:**

Bruker APEXII CCD diffractometer	4227 reflections with *I* > 2σ(*I*)
ω scans	*R*_int_ = 0.084
Absorption correction: multi-scan (*SADABS*; Bruker, 2012)	θ_max_ = 25.3°, θ_min_ = 2.5°
*T*_min_ = 0.627, *T*_max_ = 0.745	*h* = −20→20
35302 measured reflections	*k* = −16→16
7406 independent reflections	*l* = −22→22

### Refinement

**Table:**

Refinement on *F*^2^	Hydrogen site location: inferred from neighbouring sites
Least-squares matrix: full	H-atom parameters constrained
*R*[*F*^2^ > 2σ(*F*^2^)] = 0.058	*w* = 1/[σ^2^(*F*_o_^2^) + (0.0931*P*)^2^] where *P* = (*F*_o_^2^ + 2*F*_c_^2^)/3
*wR*(*F*^2^) = 0.181	(Δ/σ)_max_ = 0.001
*S* = 1.03	Δρ_max_ = 0.60 e Å^−^^3^
7406 reflections	Δρ_min_ = −0.54 e Å^−^^3^
587 parameters	Extinction correction: SHELXL-2014/7 (Sheldrick 2014, Fc^*^=kFc[1+0.001xFc^2^λ^3^/sin(2θ)]^-1/4^
598 restraints	Extinction coefficient: 0.0017 (3)
Primary atom site location: structure-invariant direct methods	

### Special details

**Table:**

Geometry. All esds (except the esd in the dihedral angle between two l.s. planes) are estimated using the full covariance matrix. The cell esds are taken into account individually in the estimation of esds in distances, angles and torsion angles; correlations between esds in cell parameters are only used when they are defined by crystal symmetry. An approximate (isotropic) treatment of cell esds is used for estimating esds involving l.s. planes.

### Fractional atomic coordinates and isotropic or equivalent isotropic displacement parameters (Å^2^)

**Table:**

	*x*	*y*	*z*	*U*_iso_*/*U*_eq_	
Ag1	0.16881 (3)	0.57600 (3)	0.42954 (2)	0.0629 (2)	
N3	0.2384 (3)	0.6205 (3)	0.5499 (2)	0.0534 (11)	
N1	0.0647 (3)	0.5949 (3)	0.3193 (2)	0.0501 (10)	
N4	0.1663 (3)	0.4387 (3)	0.5090 (2)	0.0542 (11)	
N2	0.2312 (3)	0.5968 (3)	0.3356 (2)	0.0507 (10)	
C24	0.3877 (3)	0.5755 (4)	0.4763 (3)	0.0488 (12)	
C25	0.4482 (3)	0.5944 (4)	0.5490 (3)	0.0558 (13)	
C51	0.0012 (3)	0.3458 (3)	0.2843 (3)	0.0518 (12)	
C4	0.0211 (4)	0.5902 (3)	0.1821 (3)	0.0583 (14)	
C9	0.0843 (3)	0.5942 (3)	0.2556 (3)	0.0499 (12)	
C50	0.0659 (3)	0.3530 (3)	0.3558 (3)	0.0501 (12)	
C23	0.3550 (3)	0.4777 (4)	0.4605 (3)	0.0525 (13)	
H23	0.3178	0.4627	0.4124	0.063*	
C11	0.3654 (3)	0.6531 (4)	0.4234 (3)	0.0523 (12)	
C3	−0.0622 (4)	0.5898 (4)	0.1778 (4)	0.0682 (16)	
H3	−0.1050	0.5872	0.1309	0.082*	
C35	0.2569 (3)	0.5489 (4)	0.6040 (3)	0.0514 (12)	
C52	0.0219 (3)	0.3404 (3)	0.2178 (3)	0.0553 (13)	
C2	−0.0806 (4)	0.5930 (4)	0.2405 (4)	0.0716 (17)	
H2	−0.1359	0.5941	0.2376	0.086*	
C49	0.1512 (3)	0.3484 (4)	0.3605 (3)	0.0545 (13)	
H49	0.1938	0.3495	0.4076	0.065*	
C27	0.2729 (3)	0.7083 (4)	0.5691 (3)	0.0618 (15)	
H27	0.2599	0.7572	0.5324	0.074*	
C47	0.1073 (4)	0.3406 (4)	0.2239 (3)	0.0594 (13)	
C1	−0.0154 (4)	0.5946 (4)	0.3103 (4)	0.0623 (15)	
H1	−0.0293	0.5956	0.3534	0.075*	
C21	0.4332 (3)	0.4254 (4)	0.5877 (3)	0.0593 (14)	
C8	0.1705 (3)	0.5955 (3)	0.2629 (3)	0.0512 (12)	
C26	0.4708 (3)	0.5186 (4)	0.6047 (3)	0.0568 (13)	
C22	0.3767 (3)	0.4072 (4)	0.5136 (3)	0.0593 (14)	
H22	0.3540	0.3447	0.5013	0.071*	
C38	−0.0409 (3)	0.3561 (4)	0.4127 (3)	0.0665 (16)	
H38	−0.0553	0.3579	0.4555	0.080*	
C37	0.0425 (3)	0.3630 (4)	0.4205 (3)	0.0551 (13)	
C48	0.1704 (3)	0.3424 (4)	0.2978 (3)	0.0612 (14)	
H48	0.2263	0.3393	0.3025	0.073*	
C34	0.2188 (3)	0.4562 (4)	0.5850 (3)	0.0568 (13)	
C14	0.4859 (3)	0.6873 (5)	0.5674 (3)	0.0659 (15)	
C40	−0.0837 (3)	0.3446 (4)	0.2778 (3)	0.0592 (14)	
C28	0.3280 (3)	0.7312 (5)	0.6420 (3)	0.0701 (16)	
H28	0.3504	0.7940	0.6533	0.084*	
C10	0.2978 (3)	0.6468 (4)	0.3491 (3)	0.0571 (14)	
H10	0.3042	0.6817	0.3094	0.069*	
C5	0.0460 (5)	0.5865 (4)	0.1188 (3)	0.0726 (17)	
H5	0.0057	0.5855	0.0706	0.087*	
C12	0.4057 (4)	0.7427 (4)	0.4422 (4)	0.0702 (16)	
H12	0.3930	0.7923	0.4061	0.084*	
C36	0.1001 (3)	0.3865 (4)	0.4959 (3)	0.0617 (14)	
H36	0.0884	0.3624	0.5369	0.074*	
C6	0.1272 (5)	0.5843 (4)	0.1275 (3)	0.0796 (18)	
H6	0.1419	0.5796	0.0849	0.096*	
C43	−0.0416 (4)	0.3368 (4)	0.1457 (3)	0.0679 (15)	
C39	−0.1028 (4)	0.3465 (4)	0.3430 (4)	0.0701 (16)	
H39	−0.1582	0.3413	0.3394	0.084*	
C7	0.1901 (4)	0.5889 (4)	0.1982 (3)	0.0649 (15)	
H7	0.2458	0.5877	0.2022	0.078*	
C46	0.1265 (4)	0.3377 (4)	0.1576 (3)	0.0738 (16)	
H46	0.1818	0.3394	0.1604	0.089*	
C20	0.4531 (4)	0.3517 (5)	0.6449 (4)	0.0767 (17)	
H20	0.4287	0.2898	0.6345	0.092*	
C29	0.3478 (3)	0.6613 (5)	0.6949 (3)	0.0708 (16)	
H29	0.3854	0.6753	0.7430	0.085*	
C30	0.3127 (3)	0.5674 (5)	0.6789 (3)	0.0616 (14)	
C41	−0.1471 (4)	0.3425 (4)	0.2036 (4)	0.0736 (16)	
H41	−0.2031	0.3437	0.1986	0.088*	
C42	−0.1271 (4)	0.3387 (4)	0.1413 (4)	0.0787 (17)	
H42	−0.1696	0.3374	0.0942	0.094*	
C13	0.4634 (4)	0.7603 (5)	0.5121 (4)	0.0780 (17)	
H13	0.4882	0.8220	0.5231	0.094*	
C31	0.3279 (4)	0.4912 (5)	0.7325 (3)	0.0789 (18)	
H31	0.3634	0.5023	0.7819	0.095*	
C17	0.5302 (3)	0.5385 (5)	0.6787 (3)	0.0716 (16)	
C44	−0.0196 (5)	0.3334 (4)	0.0827 (4)	0.0834 (18)	
H44	−0.0614	0.3318	0.0352	0.100*	
C18	0.5467 (4)	0.4642 (6)	0.7317 (4)	0.0867 (19)	
H18	0.5848	0.4760	0.7800	0.104*	
C19	0.5093 (4)	0.3742 (6)	0.7158 (4)	0.092 (2)	
H19	0.5219	0.3269	0.7537	0.110*	
C45	0.0616 (5)	0.3323 (5)	0.0877 (4)	0.089 (2)	
H45	0.0741	0.3279	0.0440	0.107*	
C16	0.5670 (4)	0.6338 (6)	0.6939 (4)	0.0846 (18)	
H16	0.6064	0.6475	0.7413	0.102*	
C33	0.2366 (4)	0.3821 (5)	0.6390 (3)	0.0767 (18)	
H33	0.2126	0.3202	0.6267	0.092*	
C15	0.5460 (4)	0.7034 (6)	0.6414 (4)	0.0836 (19)	
H15	0.5713	0.7647	0.6531	0.100*	
C32	0.2919 (4)	0.4028 (6)	0.7128 (4)	0.091 (2)	
H32	0.3037	0.3535	0.7491	0.109*	
O3	0.8030 (4)	0.9279 (7)	0.4993 (4)	0.201 (4)	
O2	0.6998 (7)	1.0435 (7)	0.4268 (5)	0.227 (4)	
O1	0.6838 (6)	0.8797 (8)	0.3980 (5)	0.226 (4)	
S1	0.72380 (15)	0.9402 (2)	0.45628 (13)	0.1150 (8)	
C53	0.6710 (9)	0.9393 (10)	0.5169 (9)	0.159 (4)	
F2	0.6754 (6)	0.8555 (6)	0.5521 (5)	0.248 (4)	
F1	0.6960 (7)	0.9994 (8)	0.5715 (5)	0.265 (4)	
F3	0.5898 (6)	0.9540 (8)	0.4846 (7)	0.282 (5)	

### Atomic displacement parameters (Å^2^)

**Table:**

	*U*^11^	*U*^22^	*U*^33^	*U*^12^	*U*^13^	*U*^23^
Ag1	0.0616 (3)	0.0814 (4)	0.0401 (3)	−0.0132 (2)	0.0113 (2)	0.0016 (2)
N3	0.050 (3)	0.065 (3)	0.043 (2)	−0.006 (2)	0.0139 (19)	−0.008 (2)
N1	0.051 (2)	0.042 (2)	0.054 (2)	0.0020 (18)	0.0144 (19)	−0.0029 (19)
N4	0.048 (2)	0.060 (3)	0.052 (2)	−0.0033 (19)	0.0148 (19)	0.000 (2)
N2	0.052 (2)	0.052 (3)	0.045 (2)	0.0022 (19)	0.0137 (19)	0.0049 (19)
C24	0.037 (3)	0.056 (3)	0.054 (3)	0.002 (2)	0.016 (2)	−0.002 (2)
C25	0.038 (3)	0.069 (3)	0.059 (3)	0.002 (2)	0.014 (2)	−0.007 (2)
C51	0.054 (3)	0.034 (3)	0.058 (3)	0.002 (2)	0.008 (2)	−0.005 (2)
C4	0.072 (3)	0.038 (3)	0.048 (3)	0.005 (2)	−0.001 (2)	−0.001 (2)
C9	0.055 (3)	0.044 (3)	0.040 (3)	0.003 (2)	0.004 (2)	0.004 (2)
C50	0.048 (3)	0.038 (3)	0.060 (3)	−0.003 (2)	0.015 (2)	−0.001 (2)
C23	0.043 (3)	0.054 (3)	0.057 (3)	0.004 (2)	0.014 (2)	−0.002 (2)
C11	0.043 (3)	0.055 (3)	0.061 (3)	0.001 (2)	0.021 (2)	0.001 (2)
C3	0.059 (3)	0.051 (3)	0.066 (4)	0.007 (3)	−0.012 (3)	0.002 (3)
C35	0.041 (3)	0.072 (3)	0.041 (3)	0.004 (2)	0.015 (2)	−0.006 (2)
C52	0.069 (3)	0.035 (3)	0.054 (3)	−0.006 (2)	0.013 (2)	−0.002 (2)
C2	0.058 (3)	0.053 (4)	0.085 (4)	0.002 (3)	0.003 (3)	−0.003 (3)
C49	0.046 (3)	0.056 (3)	0.057 (3)	−0.001 (2)	0.013 (2)	−0.008 (3)
C27	0.059 (3)	0.064 (3)	0.057 (3)	−0.006 (3)	0.014 (3)	−0.015 (3)
C47	0.073 (3)	0.039 (3)	0.064 (3)	−0.002 (2)	0.023 (3)	−0.005 (2)
C1	0.053 (3)	0.058 (4)	0.071 (4)	0.000 (2)	0.016 (3)	−0.007 (3)
C21	0.039 (3)	0.072 (3)	0.061 (3)	0.011 (2)	0.010 (2)	0.008 (2)
C8	0.061 (3)	0.046 (3)	0.040 (3)	0.007 (2)	0.010 (2)	0.010 (2)
C26	0.035 (3)	0.079 (3)	0.054 (3)	0.010 (2)	0.012 (2)	−0.002 (2)
C22	0.042 (3)	0.064 (3)	0.066 (3)	0.004 (2)	0.012 (2)	0.003 (3)
C38	0.055 (3)	0.069 (4)	0.078 (4)	−0.016 (3)	0.026 (3)	−0.011 (3)
C37	0.049 (3)	0.050 (3)	0.063 (3)	−0.008 (2)	0.016 (2)	−0.005 (2)
C48	0.057 (3)	0.053 (3)	0.073 (3)	0.000 (3)	0.023 (3)	−0.008 (3)
C34	0.050 (3)	0.073 (3)	0.043 (3)	0.002 (2)	0.011 (2)	0.002 (2)
C14	0.047 (3)	0.079 (4)	0.070 (3)	−0.010 (3)	0.019 (3)	−0.015 (3)
C40	0.049 (3)	0.039 (3)	0.077 (3)	−0.005 (2)	0.008 (2)	−0.005 (3)
C28	0.053 (3)	0.080 (4)	0.069 (4)	−0.007 (3)	0.011 (3)	−0.028 (3)
C10	0.060 (3)	0.054 (3)	0.058 (3)	0.003 (2)	0.022 (2)	0.009 (2)
C5	0.099 (4)	0.060 (4)	0.041 (3)	0.009 (3)	0.004 (3)	0.003 (3)
C12	0.060 (3)	0.064 (3)	0.083 (4)	−0.009 (3)	0.022 (3)	0.000 (3)
C36	0.060 (3)	0.065 (3)	0.059 (3)	−0.009 (3)	0.020 (3)	0.001 (3)
C6	0.107 (4)	0.080 (4)	0.044 (3)	0.016 (4)	0.018 (3)	0.008 (3)
C43	0.085 (4)	0.046 (3)	0.057 (3)	0.001 (3)	0.006 (3)	−0.005 (3)
C39	0.052 (3)	0.062 (4)	0.092 (4)	−0.006 (3)	0.020 (3)	−0.013 (3)
C7	0.081 (4)	0.066 (4)	0.048 (3)	0.010 (3)	0.022 (3)	0.010 (3)
C46	0.101 (4)	0.056 (4)	0.069 (4)	0.008 (3)	0.037 (3)	−0.003 (3)
C20	0.057 (4)	0.091 (4)	0.080 (4)	0.021 (3)	0.020 (3)	0.025 (3)
C29	0.051 (3)	0.097 (4)	0.055 (3)	0.002 (3)	0.007 (3)	−0.021 (3)
C30	0.045 (3)	0.091 (4)	0.043 (3)	0.007 (3)	0.009 (2)	−0.008 (2)
C41	0.053 (3)	0.057 (4)	0.089 (4)	−0.004 (3)	−0.001 (3)	−0.008 (3)
C42	0.080 (4)	0.055 (4)	0.076 (4)	0.000 (3)	−0.003 (3)	−0.007 (3)
C13	0.061 (4)	0.074 (4)	0.094 (4)	−0.019 (3)	0.021 (3)	−0.017 (3)
C31	0.064 (4)	0.111 (4)	0.048 (3)	0.007 (3)	0.002 (3)	0.005 (3)
C17	0.044 (3)	0.107 (4)	0.058 (3)	0.011 (3)	0.011 (3)	−0.011 (3)
C44	0.112 (5)	0.061 (4)	0.065 (4)	0.008 (4)	0.016 (3)	−0.002 (3)
C18	0.057 (4)	0.131 (5)	0.062 (4)	0.018 (3)	0.009 (3)	0.007 (4)
C19	0.067 (4)	0.124 (5)	0.071 (4)	0.027 (4)	0.008 (3)	0.024 (4)
C45	0.129 (5)	0.069 (4)	0.066 (4)	0.012 (4)	0.030 (4)	−0.005 (3)
C16	0.054 (4)	0.122 (5)	0.069 (4)	−0.002 (3)	0.012 (3)	−0.023 (3)
C33	0.066 (4)	0.088 (4)	0.065 (3)	−0.005 (3)	0.009 (3)	0.015 (3)
C15	0.056 (4)	0.105 (5)	0.081 (4)	−0.018 (3)	0.014 (3)	−0.030 (3)
C32	0.086 (5)	0.113 (5)	0.053 (4)	0.005 (4)	0.000 (3)	0.023 (3)
O3	0.100 (4)	0.401 (13)	0.090 (4)	0.045 (5)	0.021 (4)	0.048 (5)
O2	0.256 (10)	0.187 (6)	0.246 (10)	0.016 (6)	0.099 (8)	0.088 (6)
O1	0.201 (8)	0.253 (8)	0.170 (7)	−0.016 (6)	0.000 (6)	−0.078 (7)
S1	0.0939 (16)	0.157 (2)	0.0820 (15)	−0.0174 (15)	0.0165 (12)	−0.0017 (14)
C53	0.173 (8)	0.130 (7)	0.210 (9)	−0.030 (6)	0.112 (7)	−0.010 (6)
F2	0.316 (10)	0.199 (7)	0.310 (9)	−0.005 (6)	0.210 (8)	0.070 (6)
F1	0.336 (10)	0.268 (9)	0.281 (9)	−0.081 (7)	0.221 (8)	−0.093 (7)
F3	0.160 (6)	0.309 (10)	0.416 (12)	0.006 (6)	0.151 (7)	0.077 (8)

### Geometric parameters (Å, º)

**Table:**

Ag1—N3	2.249 (4)	C48—H48	0.9300
Ag1—N1	2.228 (4)	C34—C33	1.392 (8)
Ag1—N4	2.411 (4)	C14—C13	1.396 (8)
Ag1—N2	2.399 (4)	C14—C15	1.431 (8)
N3—C35	1.368 (7)	C40—C39	1.387 (8)
N3—C27	1.326 (6)	C40—C41	1.440 (7)
N1—C9	1.364 (7)	C28—H28	0.9300
N1—C1	1.321 (7)	C28—C29	1.336 (8)
N4—C34	1.420 (7)	C10—H10	0.9300
N4—C36	1.286 (6)	C5—H5	0.9300
N2—C8	1.400 (6)	C5—C6	1.342 (9)
N2—C10	1.274 (6)	C12—H12	0.9300
C24—C25	1.424 (7)	C12—C13	1.364 (8)
C24—C23	1.434 (7)	C36—H36	0.9300
C24—C11	1.413 (7)	C6—H6	0.9300
C25—C26	1.427 (7)	C6—C7	1.391 (8)
C25—C14	1.406 (7)	C43—C42	1.437 (9)
C51—C50	1.416 (7)	C43—C44	1.377 (9)
C51—C52	1.428 (8)	C39—H39	0.9300
C51—C40	1.417 (7)	C7—H7	0.9300
C4—C9	1.430 (7)	C46—H46	0.9300
C4—C3	1.400 (8)	C46—C45	1.395 (8)
C4—C5	1.412 (9)	C20—H20	0.9300
C9—C8	1.434 (7)	C20—C19	1.382 (9)
C50—C49	1.434 (7)	C29—H29	0.9300
C50—C37	1.426 (7)	C29—C30	1.400 (8)
C23—H23	0.9300	C30—C31	1.410 (8)
C23—C22	1.344 (7)	C41—H41	0.9300
C11—C10	1.473 (7)	C41—C42	1.344 (9)
C11—C12	1.384 (7)	C42—H42	0.9300
C3—H3	0.9300	C13—H13	0.9300
C3—C2	1.336 (9)	C31—H31	0.9300
C35—C34	1.407 (7)	C31—C32	1.343 (9)
C35—C30	1.424 (7)	C17—C18	1.382 (9)
C52—C47	1.426 (8)	C17—C16	1.427 (9)
C52—C43	1.413 (7)	C44—H44	0.9300
C2—H2	0.9300	C44—C45	1.359 (9)
C2—C1	1.396 (8)	C18—H18	0.9300
C49—H49	0.9300	C18—C19	1.366 (10)
C49—C48	1.346 (7)	C19—H19	0.9300
C27—H27	0.9300	C45—H45	0.9300
C27—C28	1.404 (7)	C16—H16	0.9300
C47—C48	1.433 (7)	C16—C15	1.327 (9)
C47—C46	1.410 (8)	C33—H33	0.9300
C1—H1	0.9300	C33—C32	1.411 (8)
C21—C26	1.406 (8)	C15—H15	0.9300
C21—C22	1.417 (8)	C32—H32	0.9300
C21—C20	1.426 (8)	O3—S1	1.321 (7)
C8—C7	1.386 (7)	O2—S1	1.515 (9)
C26—C17	1.436 (7)	O1—S1	1.352 (8)
C22—H22	0.9300	S1—C53	1.698 (11)
C38—H38	0.9300	C53—F2	1.309 (13)
C38—C37	1.387 (7)	C53—F1	1.268 (13)
C38—C39	1.375 (7)	C53—F3	1.320 (15)
C37—C36	1.454 (7)		
			
N3—Ag1—N4	72.24 (15)	C51—C40—C41	118.5 (6)
N3—Ag1—N2	120.45 (15)	C39—C40—C51	119.0 (5)
N1—Ag1—N3	151.52 (16)	C39—C40—C41	122.5 (6)
N1—Ag1—N4	119.33 (15)	C27—C28—H28	120.5
N1—Ag1—N2	73.09 (15)	C29—C28—C27	118.9 (6)
N2—Ag1—N4	131.88 (14)	C29—C28—H28	120.5
C35—N3—Ag1	117.9 (3)	N2—C10—C11	124.4 (5)
C27—N3—Ag1	123.0 (4)	N2—C10—H10	117.8
C27—N3—C35	118.3 (5)	C11—C10—H10	117.8
C9—N1—Ag1	117.5 (3)	C4—C5—H5	119.6
C1—N1—Ag1	124.6 (4)	C6—C5—C4	120.8 (6)
C1—N1—C9	117.3 (5)	C6—C5—H5	119.6
C34—N4—Ag1	111.0 (3)	C11—C12—H12	119.1
C36—N4—Ag1	121.2 (4)	C13—C12—C11	121.8 (6)
C36—N4—C34	119.0 (5)	C13—C12—H12	119.1
C8—N2—Ag1	111.0 (3)	N4—C36—C37	123.7 (5)
C10—N2—Ag1	121.2 (4)	N4—C36—H36	118.2
C10—N2—C8	120.1 (5)	C37—C36—H36	118.2
C25—C24—C23	117.8 (5)	C5—C6—H6	119.0
C11—C24—C25	118.2 (5)	C5—C6—C7	121.9 (6)
C11—C24—C23	124.0 (5)	C7—C6—H6	119.0
C24—C25—C26	119.8 (5)	C52—C43—C42	118.2 (6)
C14—C25—C24	121.0 (5)	C44—C43—C52	119.2 (6)
C14—C25—C26	119.2 (5)	C44—C43—C42	122.6 (6)
C50—C51—C52	119.6 (5)	C38—C39—C40	120.9 (6)
C50—C51—C40	120.8 (5)	C38—C39—H39	119.6
C40—C51—C52	119.6 (5)	C40—C39—H39	119.6
C3—C4—C9	117.4 (6)	C8—C7—C6	120.6 (6)
C3—C4—C5	124.1 (6)	C8—C7—H7	119.7
C5—C4—C9	118.5 (6)	C6—C7—H7	119.7
N1—C9—C4	121.6 (5)	C47—C46—H46	120.4
N1—C9—C8	119.0 (4)	C45—C46—C47	119.3 (7)
C4—C9—C8	119.4 (5)	C45—C46—H46	120.4
C51—C50—C49	119.2 (5)	C21—C20—H20	120.7
C51—C50—C37	117.9 (5)	C19—C20—C21	118.6 (7)
C37—C50—C49	122.9 (5)	C19—C20—H20	120.7
C24—C23—H23	119.2	C28—C29—H29	119.5
C22—C23—C24	121.5 (5)	C28—C29—C30	120.9 (5)
C22—C23—H23	119.2	C30—C29—H29	119.5
C24—C11—C10	123.9 (5)	C29—C30—C35	117.5 (5)
C12—C11—C24	119.5 (5)	C29—C30—C31	124.1 (6)
C12—C11—C10	116.5 (5)	C31—C30—C35	118.4 (6)
C4—C3—H3	119.8	C40—C41—H41	119.3
C2—C3—C4	120.4 (6)	C42—C41—C40	121.4 (6)
C2—C3—H3	119.8	C42—C41—H41	119.3
N3—C35—C34	118.9 (5)	C43—C42—H42	119.2
N3—C35—C30	121.1 (5)	C41—C42—C43	121.6 (6)
C34—C35—C30	120.0 (5)	C41—C42—H42	119.2
C47—C52—C51	119.9 (5)	C14—C13—H13	119.5
C43—C52—C51	120.6 (6)	C12—C13—C14	121.1 (6)
C43—C52—C47	119.5 (5)	C12—C13—H13	119.5
C3—C2—H2	120.5	C30—C31—H31	119.7
C3—C2—C1	118.9 (6)	C32—C31—C30	120.6 (6)
C1—C2—H2	120.5	C32—C31—H31	119.7
C50—C49—H49	119.6	C18—C17—C26	117.6 (6)
C48—C49—C50	120.9 (5)	C18—C17—C16	123.9 (6)
C48—C49—H49	119.6	C16—C17—C26	118.5 (6)
N3—C27—H27	118.4	C43—C44—H44	119.0
N3—C27—C28	123.2 (6)	C45—C44—C43	121.9 (7)
C28—C27—H27	118.4	C45—C44—H44	119.0
C52—C47—C48	118.3 (5)	C17—C18—H18	118.8
C46—C47—C52	119.1 (5)	C19—C18—C17	122.5 (7)
C46—C47—C48	122.6 (6)	C19—C18—H18	118.8
N1—C1—C2	124.4 (6)	C20—C19—H19	119.2
N1—C1—H1	117.8	C18—C19—C20	121.5 (7)
C2—C1—H1	117.8	C18—C19—H19	119.2
C26—C21—C22	118.7 (5)	C46—C45—H45	119.5
C26—C21—C20	119.7 (5)	C44—C45—C46	121.0 (7)
C22—C21—C20	121.6 (6)	C44—C45—H45	119.5
N2—C8—C9	118.2 (5)	C17—C16—H16	119.5
C7—C8—N2	123.0 (5)	C15—C16—C17	121.0 (6)
C7—C8—C9	118.7 (5)	C15—C16—H16	119.5
C25—C26—C17	119.8 (6)	C34—C33—H33	120.7
C21—C26—C25	120.1 (5)	C34—C33—C32	118.7 (6)
C21—C26—C17	120.0 (6)	C32—C33—H33	120.7
C23—C22—C21	121.8 (5)	C14—C15—H15	118.8
C23—C22—H22	119.1	C16—C15—C14	122.3 (7)
C21—C22—H22	119.1	C16—C15—H15	118.8
C37—C38—H38	119.3	C31—C32—C33	122.2 (6)
C39—C38—H38	119.3	C31—C32—H32	118.9
C39—C38—C37	121.4 (6)	C33—C32—H32	118.9
C50—C37—C36	124.6 (5)	O3—S1—O2	115.4 (6)
C38—C37—C50	119.7 (5)	O3—S1—O1	122.5 (6)
C38—C37—C36	115.6 (5)	O3—S1—C53	105.0 (6)
C49—C48—C47	121.9 (5)	O2—S1—C53	96.7 (6)
C49—C48—H48	119.0	O1—S1—O2	105.8 (6)
C47—C48—H48	119.0	O1—S1—C53	108.2 (7)
C35—C34—N4	118.6 (5)	F2—C53—S1	113.8 (10)
C33—C34—N4	121.2 (5)	F2—C53—F3	103.2 (10)
C33—C34—C35	120.0 (5)	F1—C53—S1	116.2 (9)
C25—C14—C15	119.1 (6)	F1—C53—F2	101.8 (13)
C13—C14—C25	118.3 (5)	F1—C53—F3	105.9 (12)
C13—C14—C15	122.6 (6)	F3—C53—S1	114.4 (12)
			
Ag1—N3—C35—C34	−11.9 (6)	C49—C50—C37—C36	11.2 (8)
Ag1—N3—C35—C30	169.1 (4)	C27—N3—C35—C34	178.1 (5)
Ag1—N3—C27—C28	−168.9 (4)	C27—N3—C35—C30	−0.9 (7)
Ag1—N1—C9—C4	170.0 (3)	C27—C28—C29—C30	−1.7 (9)
Ag1—N1—C9—C8	−8.7 (6)	C47—C52—C43—C42	−178.7 (5)
Ag1—N1—C1—C2	−170.8 (4)	C47—C52—C43—C44	0.1 (8)
Ag1—N4—C34—C35	5.4 (6)	C47—C46—C45—C44	2.4 (9)
Ag1—N4—C34—C33	−170.8 (4)	C1—N1—C9—C4	−1.9 (7)
Ag1—N4—C36—C37	36.1 (7)	C1—N1—C9—C8	179.4 (4)
Ag1—N2—C8—C9	7.1 (5)	C21—C26—C17—C18	−1.5 (8)
Ag1—N2—C8—C7	−168.5 (4)	C21—C26—C17—C16	180.0 (5)
Ag1—N2—C10—C11	33.2 (7)	C21—C20—C19—C18	−1.1 (10)
N3—C35—C34—N4	3.7 (7)	C8—N2—C10—C11	−179.9 (5)
N3—C35—C34—C33	179.9 (5)	C26—C25—C14—C13	179.6 (5)
N3—C35—C30—C29	0.0 (8)	C26—C25—C14—C15	0.2 (8)
N3—C35—C30—C31	178.8 (5)	C26—C21—C22—C23	−3.5 (8)
N3—C27—C28—C29	0.7 (9)	C26—C21—C20—C19	−0.1 (9)
N1—C9—C8—N2	0.4 (7)	C26—C17—C18—C19	0.3 (10)
N1—C9—C8—C7	176.3 (4)	C26—C17—C16—C15	1.2 (9)
N4—C34—C33—C32	177.4 (6)	C22—C21—C26—C25	3.6 (8)
N2—C8—C7—C6	177.6 (5)	C22—C21—C26—C17	−178.2 (5)
C24—C25—C26—C21	−0.2 (8)	C22—C21—C20—C19	179.4 (6)
C24—C25—C26—C17	−178.4 (5)	C38—C37—C36—N4	−146.2 (6)
C24—C25—C14—C13	−0.9 (8)	C37—C50—C49—C48	−177.7 (5)
C24—C25—C14—C15	179.7 (5)	C37—C38—C39—C40	−0.8 (9)
C24—C23—C22—C21	−0.2 (8)	C48—C47—C46—C45	177.5 (5)
C24—C11—C10—N2	32.7 (8)	C34—N4—C36—C37	−179.3 (5)
C24—C11—C12—C13	−3.6 (9)	C34—C35—C30—C29	−179.1 (5)
C25—C24—C23—C22	3.6 (7)	C34—C35—C30—C31	−0.2 (8)
C25—C24—C11—C10	−173.5 (5)	C34—C33—C32—C31	−0.3 (11)
C25—C24—C11—C12	3.3 (7)	C14—C25—C26—C21	179.3 (5)
C25—C26—C17—C18	176.7 (5)	C14—C25—C26—C17	1.1 (8)
C25—C26—C17—C16	−1.8 (8)	C40—C51—C50—C49	176.5 (4)
C25—C14—C13—C12	0.8 (9)	C40—C51—C50—C37	−2.4 (7)
C25—C14—C15—C16	−0.8 (9)	C40—C51—C52—C47	−179.4 (4)
C51—C50—C49—C48	3.5 (7)	C40—C51—C52—C43	1.8 (7)
C51—C50—C37—C38	6.0 (7)	C40—C41—C42—C43	0.0 (9)
C51—C50—C37—C36	−169.9 (5)	C28—C29—C30—C35	1.4 (8)
C51—C52—C47—C48	2.4 (7)	C28—C29—C30—C31	−177.4 (6)
C51—C52—C47—C46	−178.5 (5)	C10—N2—C8—C9	−142.8 (5)
C51—C52—C43—C42	0.1 (8)	C10—N2—C8—C7	41.5 (7)
C51—C52—C43—C44	178.9 (5)	C10—C11—C12—C13	173.4 (6)
C51—C40—C39—C38	4.4 (8)	C5—C4—C9—N1	−178.0 (5)
C51—C40—C41—C42	1.9 (8)	C5—C4—C9—C8	0.7 (7)
C4—C9—C8—N2	−178.3 (4)	C5—C4—C3—C2	179.7 (5)
C4—C9—C8—C7	−2.5 (7)	C5—C6—C7—C8	0.4 (9)
C4—C3—C2—C1	−1.4 (8)	C12—C11—C10—N2	−144.2 (6)
C4—C5—C6—C7	−2.2 (9)	C36—N4—C34—C35	−142.6 (6)
C9—N1—C1—C2	0.5 (7)	C36—N4—C34—C33	41.3 (8)
C9—C4—C3—C2	0.0 (7)	C43—C52—C47—C48	−178.8 (5)
C9—C4—C5—C6	1.6 (8)	C43—C52—C47—C46	0.3 (7)
C9—C8—C7—C6	2.0 (8)	C43—C44—C45—C46	−2.0 (10)
C50—C51—C52—C47	1.1 (7)	C39—C38—C37—C50	−4.5 (8)
C50—C51—C52—C43	−177.7 (4)	C39—C38—C37—C36	171.7 (5)
C50—C51—C40—C39	−2.7 (7)	C39—C40—C41—C42	−178.7 (6)
C50—C51—C40—C41	176.7 (5)	C46—C47—C48—C49	177.8 (5)
C50—C49—C48—C47	0.2 (8)	C20—C21—C26—C25	−176.9 (5)
C50—C37—C36—N4	29.9 (9)	C20—C21—C26—C17	1.3 (8)
C23—C24—C25—C26	−3.4 (7)	C20—C21—C22—C23	177.0 (5)
C23—C24—C25—C14	177.1 (5)	C29—C30—C31—C32	180.0 (6)
C23—C24—C11—C10	8.4 (8)	C30—C35—C34—N4	−177.2 (5)
C23—C24—C11—C12	−174.8 (5)	C30—C35—C34—C33	−1.0 (8)
C11—C24—C25—C26	178.4 (5)	C30—C31—C32—C33	−1.0 (11)
C11—C24—C25—C14	−1.1 (8)	C41—C40—C39—C38	−175.0 (5)
C11—C24—C23—C22	−178.3 (5)	C42—C43—C44—C45	179.5 (6)
C11—C12—C13—C14	1.5 (10)	C13—C14—C15—C16	179.8 (6)
C3—C4—C9—N1	1.7 (7)	C17—C18—C19—C20	0.9 (11)
C3—C4—C9—C8	−179.6 (4)	C17—C16—C15—C14	0.1 (10)
C3—C4—C5—C6	−178.1 (5)	C44—C43—C42—C41	−179.7 (6)
C3—C2—C1—N1	1.2 (8)	C18—C17—C16—C15	−177.3 (6)
C35—N3—C27—C28	0.6 (8)	C16—C17—C18—C19	178.8 (6)
C35—C34—C33—C32	1.3 (9)	C15—C14—C13—C12	−179.9 (6)
C35—C30—C31—C32	1.2 (9)	O3—S1—C53—F2	−63.8 (13)
C52—C51—C50—C49	−4.0 (7)	O3—S1—C53—F1	53.9 (14)
C52—C51—C50—C37	177.1 (4)	O3—S1—C53—F3	177.8 (10)
C52—C51—C40—C39	177.8 (5)	O2—S1—C53—F2	177.6 (12)
C52—C51—C40—C41	−2.7 (7)	O2—S1—C53—F1	−64.7 (14)
C52—C47—C48—C49	−3.1 (8)	O2—S1—C53—F3	59.3 (11)
C52—C47—C46—C45	−1.5 (8)	O1—S1—C53—F2	68.5 (14)
C52—C43—C42—C41	−1.0 (8)	O1—S1—C53—F1	−173.7 (12)
C52—C43—C44—C45	0.8 (9)	O1—S1—C53—F3	−49.8 (12)
C49—C50—C37—C38	−172.9 (5)		

### Hydrogen-bond geometry (Å, º)

**Table:**

*D*—H···*A*	*D*—H	H···*A*	*D*···*A*	*D*—H···*A*
C3—H3···O3^i^	0.93	2.44	3.359 (10)	140

Symmetry code: (i) *x*−1, −*y*+3/2, *z*−1/2.
